# The Impact of Stimulus Valence and Emotion Regulation on Sustained Brain Activation: Task-Rest Switching in Emotion

**DOI:** 10.1371/journal.pone.0093098

**Published:** 2014-03-28

**Authors:** Jan-Peter Lamke, Judith K. Daniels, Denise Dörfel, Michael Gaebler, Rasha Abdel Rahman, Falk Hummel, Susanne Erk, Henrik Walter

**Affiliations:** 1 Division of Mind and Brain Research, Department of Psychiatry & Psychotherapy, Charité – Universitätsmedizin Berlin, Berlin, Germany; 2 Department of Psychology, Humboldt University of Berlin, Berlin, Germany; 3 Clinic for Psychosomatic Medicine and Psychotherapy, Otto-von-Guericke University Magdeburg, Magdeburg, Germany; 4 Department of Neurology, Max Planck Institute for Human Cognitive and Brain Sciences, Leipzig, Germany; 5 Berlin School of Mind and Brain, Humboldt University of Berlin, Berlin, Germany; 6 Division of Medical Psychology, Department of Psychiatry and Psychotherapy, University of Bonn, Bonn, Germany; University Medical Center Goettingen, Germany

## Abstract

Task-rest interactions, defined as the modulation of brain activation during fixation periods depending on the preceding stimulation and experimental manipulation, have been described repeatedly for different cognitively demanding tasks in various regions across the brain. However, task-rest interactions in emotive paradigms have received considerably less attention. In this study, we therefore investigated task-rest interactions evoked by the induction and instructed regulation of negative emotion. Whole-brain, functional MRI data were acquired from 55 healthy participants. Two-level general linear model statistics were computed to test for differences between conditions, separately for stimulation and for fixation periods, as well as for interactions between stimulation and fixation (task-rest interactions). Results showed that the regulation of negative emotion led to reverse task-rest interactions (decreased activation during stimulation but increased activation during fixation) in the amygdala as well as in visual cortex regions and to concordant task-rest interactions (increased activation during both, stimulation and fixation) in the dorsolateral prefrontal cortex as well as in a number of brain regions at the intersection of the default mode and the dorsal attention networks. Thus, this first whole-brain investigation of task-rest interactions following the induction and regulation of negative emotion identified a widespread specific modulation of brain activation in regions subserving emotion generation and regulation as well as regions implicated in attention and default mode.

## Introduction

### 1.1 General introduction

In a recent review, Northoff and colleagues [1] introduced the concept of task-rest interactions, defined as the modulation of resting brain activation by the preceding stimulus-induced activation. Converging findings show that task-rest interactions are not due simply to carry-over effects or undershoot. Task-rest interactions been observed at different time scales [2], [3] and, following emotional stimulation, could not be explained by a non-specific carry-over of sensory-driven BOLD changes [5]. They have also been found in various regions across the brain [6], [7] whereas activation-dependent undershoots after stimulus offset have only been described for visual brain regions [4]. Importantly, in line with these findings, a recent study by Mullinger and colleagues [8] has reinforced that poststimulus BOLD responses represent distinct neuronal processes which did not mechanistically follow the primary activation. Task-rest interactions have been demonstrated repeatedly for different cognitively demanding tasks (e.g. [6]–[7]) while the modulatory effect of preceding *affective* stimuli (e.g. [5]) has received considerably less attention. The present study therefore aimed at analyzing task-rest interactions evoked by the induction of negative emotion and its active, instructed regulation. The inclusion of a condition during which subjects deploy a previously trained emotion regulation strategy allows for an experimental manipulation of the perceived valence of the stimuli. Gross [9] distinguished five classes of explicit, intentional emotion regulation strategies, three of which (reappraisal, response modulation, and attentional deployment) were used in the present experiment (also see [10]). The inclusion of an emotion regulation condition seems particularly important, as previous studies demonstrated aberrations during the resting state in a wide range of mental disorders characterized by pathological emotion processing and deficient emotion regulation [11], [12]. It is thus necessary to lay the foundations for future studies investigating whether similar aberrations can be observed in the switching from emotion-related tasks to rest in patient populations.

As emotion-related phenomena typically do not show a long time lag (e.g. compared to longer-lasting effects of stress or cognitive training), it is advisable to evaluate a high number of rapid task-rest switches (as opposed to the switching from a task to one long resting-state) in order to characterize the switching process. Therefore, fixation periods in cognitively or emotionally demanding experiments lend themselves to such analyses. Fair et al. [13] demonstrated that the functional connectivity during interleaved fixation periods taken from blocked designs was both qualitatively and quantitatively very similar to that of continuous resting-state data. As rapid task-rest switches frequently happen in real-world settings [14], the analysis of rapid task-rest interactions will further increase the ecological validity of such analyses.

### 1.2 Task-rest interactions

A sensible approach for classifying task-rest interactions is to differentiate between effects on task-positive regions typically showing *activation* during tasks and task-negative regions typically showing *deactivation* during tasks [1]. Spontaneous resting-state activation is assumed to be low in task-positive regions and high in task-negative regions which form the default mode network (DMN; [15]–[16]). However, both task-positive and task-negative brain regions can be recruited by specific types of tasks like working memory and self-referential processing, respectively, and the above-described differentiation is therefore mainly heuristic in nature.

Recent research has highlighted the significance of the suppression of DMN activation during tasks that require executive functions, indicating that a fluent interplay of task-positive and task-negative regions is a prerequisite for goal-directed cognition [17]. Convergently, DMN suppression deficits have been reported in severe mental illness, linking disorder-specific cognitive difficulties to insufficient switching from task to rest and vice versa ([18]–[19], for a review see [20]).

### 1.3 Task-rest interactions in emotive paradigms

To date, five studies are available which demonstrated modulatory effects of preceding *emotive* stimuli on activation and functional connectivity of *task-negative* regions (DMN) during interspersed fixation blocks [5] [21]–[24]. Typically, these studies employed experiments which consisted of task blocks (during which either neutral or affective stimuli were presented) alternating with fixation blocks. In order to extract the modulatory effects of the preceding stimulation, brain activation and functional connectivity during fixation periods following affective stimuli were compared with activation and connectivity during fixation periods following neutral stimuli.

While Pitroda et al. [23] reported attenuated DMN activation and Harrison et al. [22] observed decreased functional connectivity between DMN regions following affective stimulation, Schneider et al. [24] reported increased activation of prefrontal and posterior DMN regions. Eryilmaz et al. [5] in turn showed attenuated DMN activation while functional connectivity was enhanced within the DMN as well as between left insula and DMN regions and reduced between bilateral amygdala and DMN regions.

The only study to date which explicitly focused on task-rest interactions in a *task-positive* brain region reported activation differences in the amygdala. Walter et al. [25] demonstrated that the effect of instructed emotion regulation on amygdala activation extended beyond the regulation period itself in the form of a paradoxical rebound effect: while amygdala activation was effectively reduced during regulation, it subsequently increased during the following fixation period.

### 1.4 Aims of the study

Following Walter et al. [25] and extending their approach to the whole brain, the present fMRI study aims at understanding how induction and instructed regulation of negative emotion modulate brain activation during subsequent rest periods.

Differential brain activations during rest can only be interpreted as task-rest interactions in relation to differential brain activation during the preceding tasks. Therefore, only regionally specific task-rest interactions are considered for the present analysis. That is, a statistically significant difference in the activation of a brain region between two experimental conditions during rest is understood as a task-rest interaction only if that same brain region also shows a difference in activation between the two conditions during the preceding task period. Task-rest interactions may either be *concordant*, that is, condition 1 activating a brain region more strongly than condition 2 during both stimulation and fixation, or *reverse*, that is, condition 1 activating a brain region more strongly than condition 2 during stimulation but condition 2 activating it more strongly than condition 1 during fixation.

In order to specifically test for the presence of such task-rest interactions, we analyzed data from an fMRI experiment on emotion regulation, the task-related results of which are being published elsewhere (Dörfel et al., under review). The data were subjected to preprocessing and then entered into a two-level general linear model analysis using SPM. Differences between experimental conditions were examined separately for stimulation and for fixation/rest periods as well as for task-rest interactions between stimulation and fixation.

Based on our previous publication [25], we expected the above-described task-rest interactions particularly in the bilateral amygdala. In addition, emotion regulation was hypothesized to elicit task rest interactions in regions involved in top-down regulatory control such as medial and lateral prefrontal cortex and dorsal anterior cingulate cortex, as well as intermediary regions subserving stimulus appraisal and reappraisal such as the orbitofrontal cortex and temporal lobe structures [10] [26]–[27].

## Methods

### 2.1 Participants and Ethics Statement

The study was approved by the ethics committee of the University of Bonn and was conducted in accordance with the principles expressed in the Declaration of Helsinki. The participants' written informed consent was obtained and they received a monetary reward for their participation. Eighty-three healthy female participants were recruited in Bonn, Germany. Data from nine participants had to be excluded from further analyses due to movement artifacts or technical problems. Data from 74 participants were then included in the preprocessing. Participants were assigned to four groups instructed to use different emotion regulation strategies, one of which (reinterpretation) showed a markedly different brain activation pattern (Dörfel et al., under review). As our study aims at investigating task-rest interactions following emotion induction and regulation rather than strategy-specific activation patterns, this group was excluded from the present investigation. In total, data from 55 participants were analyzed (age M = 23.36, SD = 3.60); there were no significant age differences between the three groups (F[2, 52] = 0.42, p = 0.66). Before the scan, participants were trained to deploy one of the following three emotion regulation strategies during the ‘regulate’ condition: detaching themselves by taking the position of a non-involved observer (reappraisal/distancing; 17 participants), suppressing any facial expression of emotion (response modulation; 22 participants), or distracting themselves by remembering a previously learned 9-digit number (attentional deployment; 16 participants). For the present analysis, data from detachment, expressive suppression, and distraction groups were pooled together; task-fMRI results for all groups as well as for group differences are reported by Dörfel et al. (under review).

### 2.2 Functional MRI task

The task employed in the present experiment is well-established and has been successfully used in previous studies [25] [28]–[29]. The experimental paradigm was divided into four runs, each consisting of 15 trials. In each trial, participants were presented with a picture with either aversive or neutral content. Participants were instructed to either naturally experience (‘permit’ condition) or to actively down-regulate (‘regulate’ condition) their emotional responses to the presented stimuli. Equal numbers of aversive pictures were presented with the ‘permit’ instruction and with the ‘regulate’ instruction while neutral pictures were only presented with the ‘permit’ instruction.

Thus, in each run, 10 aversive pictures (5× regulate, 5× permit) and 5 neutral pictures (permit only) were presented, totaling 20 stimuli per condition (regulated aversive, unregulated aversive, unregulated neutral) across all four runs. Pictures were taken from a standardized stimulus set (IAPS, 30; demonstrated to reliably elicit emotional responses in a German sample, 31), matched for complexity, content, color, and brightness. Each stimulus was presented for 8 s, preceded by a 2 s presentation of a German instruction word, that is, “zulassen” (permit) or “regulieren” (regulate), and followed by a 14 s fixation/rest period (white fixation cross on black background), during which participants were instructed to relax. Trial order within each run was identical for all participants and pseudo-randomized (taking into account experimental conditions and picture content) with a maximum of two consecutive trials of the same condition. In-between the runs, participants were given short breaks (according to their need) within the scanner in order to prevent increasing fatigue over the course of the experiment.

After the scanning session, valence ratings for each of the 60 pictures were obtained from 42 of the 55 participants and aggregate post-scan ratings of regulation success and of preoccupation with the preceding pictures during fixation were obtained from 46 of the 55 participants. Due to technical reasons, ratings could not be collected from all participants. A comprehensive set of behavioral measures is provided elsewhere (Dörfel et al., under review). Specifically, participants were asked to provide the following ratings on 9-point Likert-type scales: valence ratings (1 = very unpleasant to 9 = very pleasant) for each of the 60 pictures which were presented again in pseudorandomized order (without regulation instructions) as well as aggregate ratings of overall regulation success (for the whole experiment; 1 = not successful to 9 = very successful), overall perceived usefulness of the instructed regulation strategy (for the whole experiment; 1 = not helpful to 9 = very helpful), overall compliance with the instructed regulation strategy (for each run; 1 = never used it to 9 = always used it) and preoccupation with the preceding pictures during fixation per experimental condition (for the whole experiment; 1 = not preoccupied to 9 = strongly preoccupied).

### 2.3 Data Acquisition

Presentation (Neurobehavioral Systems, Albany, CA, USA) was used for stimulus delivery. MRI was performed on a 3.0 Tesla Siemens Trio scanner (Siemens AG, Erlangen, Germany) with a standard 12-channel head coil at the University of Bonn. Visual stimuli were presented via binocular video goggles (NNL, Bergen, Norway), which were attached to the head coil and adjusted to fit each participant's vision.

A high-resolution T1-weighted anatomical image was acquired at the beginning of each scanning session. BOLD fMRI data were collected using a T2*-weighted gradient-echo echo-planar imaging sequence. Acquisition parameters were: field of view = 192 mm, matrix = 64×64 pixels, repetition time (TR) = 2 s, echo time (TE) = 30 ms, flip angle = 80°. Thirty-four axial slices with a thickness of 3.0 mm and a 25% gap (voxel size = 3.0 mm×3.0 mm×3.75 mm) covering the whole brain were acquired in ascending order. The slices were axially tilted by −45°, following an imaginary line from the lower boundary of the orbitofrontal cortex to the lower boundary of the cerebellar nodule.

### 2.4 Imaging data preprocessing

Image preprocessing and statistical analyses were carried out using SPM8 (Functional Imaging Laboratory, Wellcome Trust Centre for Neuroimaging, Institute of Neurology, UCL, www.fil.ion.ucl.ac.uk/spm) and Matlab v 7.10.0.499 (R2010a, The MathWorks, Inc., Natick, Massachusetts, USA). Detailed descriptions of all processing steps are given in the SPM8 manual (http://www.fil.ion.ucl.ac.uk/spm/doc/manual.pdf). For each participant, functional scans were realigned to the first image of the first run in order to adjust for head motion (based on [32]–[33]) and the structural image was coregistered [34] to the mean functional image generated during realignment. The realigned images were corrected for differences in acquisition times across slices. Rigidly aligned tissue-class images for gray and white matter and cerebrospinal fluid were generated from the coregistered T1 images employing the ‘New Segment’ function (an extension of [35]). Rigidly aligned (‘DARTEL imported’) tissue-class images are separate images of different types of brain tissue that are ready to use with the DARTEL toolbox included in SPM8 ([36], see also SPM8 manual). Based on these images, structural templates for the whole group and individual flow fields (storing the deformation information for warping each participant's images onto the final template) were created using the DARTEL algorithm [36]. The normalization function of the SPM8 DARTEL toolbox was then employed to normalize the functional images to MNI space (using templates and flow fields from the previous step), reslice them to 3 mm isovoxels, and smooth the images with an 8-mm full-width at half maximum (FWHM) isotropic Gaussian kernel.

### 2.5 Statistical processing

Preprocessed functional images were analyzed using a two-level general linear model approach [37]–[38]. In short, the general linear model analysis in SPM8 is based on the construction of a design matrix for each participant containing a set of regressors that represent hypothesized contributors to the BOLD signal measured in a given experiment.

Parameter weights representing how strongly the regressors match changes in the measured signal are estimated for each regressor in each voxel. On the first level, hypotheses are then tested by evaluating whether the experimental manipulation caused a significant change in the parameter weights (i.e., by computing contrasts between certain conditions). On the second-level, within- and between-group comparisons can be computed based on the first-level results.

The evoked blood oxygen level-dependent (BOLD) responses for all conditions of the experimental paradigm were modeled as seven separate regressors (regulated aversive stimulation, unregulated aversive stimulation, unregulated neutral stimulation, fixation following regulated aversive stimulation, fixation following unregulated aversive stimulation, fixation following unregulated neutral stimulation, instruction) by convolving boxcar functions (step functions which are zero except for a single interval where they have a constant non-zero value) with a canonical hemodynamic response function (see SPM8 manual and [37]). Head movement parameters were included as regressors of no interest. Intrinsic autocorrelations were accounted for by a 1st-order autoregressive model and low frequency drifts were removed by applying a high-pass filter (128 sec, see SPM8 manual and [37]).

A binarized SPM8 a priori brain mask thresholded at 0.4 was used to mask first-level SPM statistics. On the first level, differential contrasts were created by contrasting the regulated aversive with unregulated aversive condition as well as the regulated aversive with the neutral condition, separately for stimulation (e.g., regulated aversive stimulation (Stim_RegAv)>unregulated aversive stimulation (Stim_Av)) as well as for fixation periods (e.g., fixation following regulated aversive stimulation (Fix_RegAv)>fixation following unregulated aversive stimulation (Fix_Av)). The regulated aversive and the neutral condition were not contrasted with each other as this contrast, confounding emotion and regulation conditions, would most likely show amalgamated emotion and regulation effects and therefore be effectively uninterpretable.

On the second level, the images of the differential first-level contrasts were entered into separate one-sample t-tests to compute the according group statistics and into paired t-tests in order to test for interactions (e.g., [Stim_RegAv>Stim_Av]>[Fix_Av>Fix_RegAv] for the concordant task-rest interaction of regulated aversive stimulation>unregulated aversive stimulation; [Stim_Av>Stim_RegAv]>[Fix_Av>Fix_RegAv] for the reverse task-rest interaction of unregulated aversive stimulation>regulated aversive stimulation). Detailed examples for the modeling of the interactions are given in Methods Text S1.

Results were thresholded at p<0.05 family-wise error (FWE, [39]) corrected for multiple comparisons across the whole brain and in left and right amygdala regions of interest (ROIs) defined according to the AAL atlas implemented in the WFU PickAtlas v3.0 [40]–[42].

Activation in one of the above-described paired t-tests (i.e., interaction contrasts) is only interpreted as a task-rest interaction if the brain regions that are activated in the paired t-test also show significant activation in the second-level one-sample t-tests on the stimulation contrasts and the fixation contrasts that are being tested for interaction. Thus, the intersection maps of the activations in the respective second-level one-sample t-tests for both the stimulation and the fixation condition were used to mask the statistical parametric maps resulting from each paired t-test. As the amygdala, which is of particular interest in the present analysis, is a comparatively small structure, we created intersection maps from activations observed in separate left and right amygdala ROI analyses (FWE-corrected) to specifically detect task-rest interactions in the amygdala. Examples for the creation of the masks are given in the Supporting Information materials. Full details on activated brain regions are provided in Table 1 and Table S1.

**Table 1:** Task-rest interactions of regulated aversive and unregulated aversive stimulation.

Contrast	Region	Right/left	Cluster size (voxels)	t-score local max.	MNI coordinates (x, y, z)		
[Stim_RegAv>Stim_Av]>[Fix_Av>Fix_RegAv]	inferior parietal lobule	R	386	13.02	51	−54	48
	middle frontal gyrus	R	460	11.66	39	48	18
	middle frontal gyrus	R		11.36	39	30	39
	middle frontal gyrus	R		10.11	33	42	33
	precuneus	R	75	9.89	9	−66	39
	precuneus	L		7.98	−3	−72	42
	middle cingulate cortex	R	82	9.69	3	−21	30
	inferior parietal lobule	L	159	9.45	−42	−60	51
	inferior parietal lobule	L		8.25	−54	−54	42
	middle frontal gyrus	L	9	7.84	−36	48	15
	precuneus	L	1	7.72	−6	−66	36
	cerebellum	L	15	7.25	−39	−48	−42
	middle frontal gyrus	R	1	7.03	24	57	21
	middle cingulate cortex	R	3	6.93	6	30	36
	inferior frontal operculum	R	3	6.85	51	15	3
[Stim_Av>Stim_RegAv]>[Fix_Av>Fix_RegAv]	superior parietal lobule	R	11	9.07	30	−51	63
	cuneus	R	26	8.51	9	−99	15
	lingual gyrus	R	29	8.37	18	−87	−9
	fusiform gyrus	R		7.70	27	−81	−9
	fusiform gyrus	L	7	7.29	−27	−75	−18
	inferior temporal gyrus	R	2	7.26	54	−54	−18
	calcarine fissure	L	16	7.22	−6	−96	9
	middle occipital gyrus	R	8	7.07	27	−84	18
	middle occipital gyrus	L	2	6.52	−24	−87	18
	amygdala	R	20	8.37	24	−3	−18
	amygdala	L	20	8.16	−30	−3	−21
	amygdala	L		7.66	−21	−6	−18
	amygdala	L		5.70	−15	0	−15

**Figure 1:**
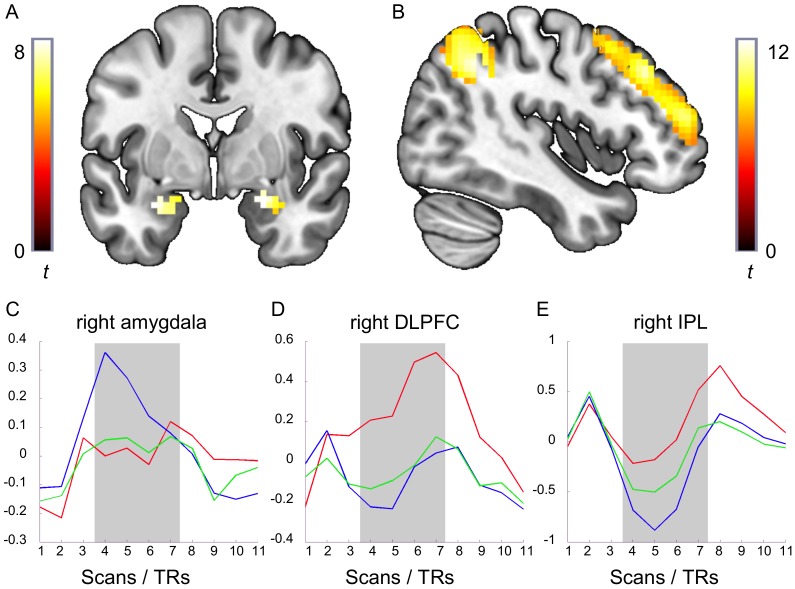
Reverse and concordant task-rest interactions. The figure depicts activated clusters (whole-brain FWE-corrected) for the reverse task-rest interaction in the amygdala (A) and the concordant task-rest interaction in DLPFC and IPL (B) – the latter showing a representative time course for task-negative regions. Peak coordinates for these clusters can be obtained from Table 1. In addition, it shows the graphs of the grand mean right amygdala (C), right DLPFC (D), and right IPL (E) time courses (computed over blocks and participants) for regulated (red) and unregulated aversive (blue) and unregulated neutral (green) stimulation-fixation. Stimulation onset is at TR1, stimulation offset is at TR 4. The activation in response to the stimulation (shaded in gray) should be expected to be delayed by about 3 TRs which corresponds to the typical lag of the canonical hemodynamic response.

The table shows anatomical labels, cluster sizes, t-scores, and coordinates in MNI space for brain activations in the contrasts of interest; threshold: p<.05, FWE-corrected. Stim_RegAv = regulated aversive stimulation; Stim_Av = unregulated aversive stimulation; Fix_RegAv = fixation following regulated aversive stimulation; Fix_Av = fixation following unregulated aversive stimulation. The main results are visualized in Figure 1.

Behavioral effects were analyzed using SPSS Statistics 17.0 (SPSS Inc., Chicago, USA). For each participant, mean valence ratings were computed per experimental condition and used for further testing. (Sub-)group means and standard deviations were computed for all ratings. Analyses of variance (ANOVAs) and post-hoc t-tests were computed to test for significant differences (p<0.05) between experimental conditions and between regulation strategy groups. Correlations were computed for regulation success, compliance, and usefulness of the strategy.

### 2.6 Complementary time course plots

In order to facilitate the interpretation of task-rest interactions, for each subject, we extracted the averaged 1st eigenvariate time series adjusted for effects of interest from brain regions showing a significant task-rest interaction in the second-level GLM analysis, that is, activation in one of the paired t-tests (interaction contrasts). Time series were extracted from a sphere with a 5-mm radius centered around the respective aftereffect clusters' centers of mass (nearest voxel) derived from the following contrasts:

[Stim_Av>Stim_RegAv]>[Fix_Av>Fix_RegAv] resulting in amygdala (x,y,z = [−24,−3,−21]; [27,−3,−21]), postcentral gyrus (x,y,z = [30,−51,63]), and early visual areas (x,y,z = [12,−99,12]; [−12,−96,9]; [22,−84,−9];[27,−84,21]) time courses
[Stim_RegAv>Stim_Av]>[Fix_Av>Fix_RegAv] resulting in dorsolateral prefrontal cortex (x,y,z = [−36,48,15]; [39], [36], [30]), inferior parietal lobule (x,y,z = [−48,−57,45]; [48,−51,45]), posterior cingulate cortex/posterior midcingulate cortex (x,y,z = [3 −30 30]), and precuneus (x,y,z = [9 −66 42]) time courses
[Stim_Av>Stim_Neu (neutral stimulation)]>[Fix_Av>Fix_Neu (following neutral stimulation)] resulting in visual area V5/MT (x,y,z = [−45 −72 6]; [45 −63 3]) time courses.

As a qualitative verification of aftereffects found with the GLM analysis, the time series from all subjects were averaged and plotted (see Figure 1 and Supporting information materials).

## Results

### 3.1 Behavioral measures

A repeated measures ANOVA indicated a significant main effect of valence (F[1.32, 39] = 351.79, p<.001, Greenhouse-Geisser corrected), but no significant interaction with emotion regulation strategy (F[2.64, 78] = 0.76, p = .509, Greenhouse-Geisser corrected). Post-hoc paired samples t-tests revealed that both, formerly regulated (M = 3.28, SD = 0.56; T[41] = −18.73, p<.001) and unregulated aversive stimuli (M = 3.01, SD = 0.54; T[41] = −20.86, p<.001), were rated as significantly less pleasant than neutral stimuli (M = 5.70, SD = 0.64). Moreover, significant differences in the valence ratings emerged between formerly regulated aversive stimuli and unregulated aversive stimuli (T[41] = −4.66, p<.001).

The post-scan ratings of overall emotion regulation success, overall compliance with the instructed regulation strategy, and overall perceived usefulness of the strategy for the experiment as a whole indicated successful emotion regulation (M = 6.78, SD = 1.11) without significant differences between regulation strategies (F[2, 43] = 0.75, p = .478; indicated by a one-way ANOVA), high compliance with the instructed strategy (M = 8.65, SD = 1.06) also without significant differences between regulation strategies (F[2, 43] = 1.41, p = .256; indicated by a one-way ANOVA), and ample usefulness of the strategy (M = 5.87, SD = 1.92), whereby not all strategies were perceived as equally useful (F[2, 43] = 5.91, p = .005; indicated by a one-way ANOVA). Post-hoc independent samples t-tests revealed that both, detachment (M = 6.47, SD = 1.85; T[31] = 2.65, p = .012) and distraction (M = 6.69, SD = 1.49; T[29] = 3.13, p = .004), were perceived as significantly more useful than expressive suppression (M = 4.78, SD = 1.80).

A repeated measures ANOVA indicated a significant main effect of preoccupation with the preceding pictures during fixation (F[1.42, 43] = 54.19, p<.001, Greenhouse-Geisser corrected) but no significant interaction with emotion regulation strategy (F[2.85, 78] = 1.85, p = .151, Greenhouse-Geisser corrected). Post-hoc paired samples t-tests revealed no significant difference (T[45] = −1.44, p = .16) between regulated (M = 5.48, SD = 2.32) and unregulated (M = 5.80, SD = 2.24) aversive stimulation, while the neutral pictures (M = 2.59, SD = 2.21) elicited significantly less preoccupation than regulated aversive (T[45] = 7.61, p<.001) and unregulated aversive (T[45] = 8.11, p<.001) stimuli.

Overall emotion regulation success neither correlated with overall compliance (r[46] = .142, p = .347) nor with overall perceived usefulness of the instructed strategy (r[46] = .091, p = .550) and compliance and usefulness were also uncorrelated (r[46] = −.023, p = .880).

### 3.2 Concordant task-rest interactions

A concordant task-rest interaction is defined as condition 1 activating a brain region more strongly than condition 2 during both stimulation and fixation.

Concordant task-rest interactions of regulated aversive stimulation>unregulated aversive stimulation (contrast: [Stim_RegAv>>Stim_Av]>[Fix_Av>Fix_RegAv]) were found in the bilateral inferior parietal lobule, bilateral dorsolateral prefrontal cortex, bilateral precuneus, bilateral posterior and middle cingulum, and left cerebellum (see Figure 1 and Table 1). Thus, these regions exhibit increased activation not only during stimulation but also during the following fixation period. No further concordant task-rest interactions were observed. Representative time courses with detailed consideration of the curve shapes are depicted in Figure 1 and the full set of time courses is provided in the Supporting Information materials to illustrate this task-rest interaction.

The dorsolateral prefrontal cortex and the bilateral inferior parietal lobule, posterior and middle cingulate cortex, and precuneus regions exhibiting a concordant task-rest interaction following regulated aversive stimulation>unregulated aversive stimulation lie precisely at the intersection of the DMN and the dorsal attention network (DAN, [43]) as extracted by Tomasi and Volkow [44].

Posthoc analyses showed no significant differences in the observed concordant task-rest interactions between the emotion regulation strategies employed (see limitations section).

### 3.3 Reverse task-rest interactions

A reverse task-rest interaction is defined as condition 1 activating a brain region more strongly than condition 2 during stimulation and vice versa during fixation.

Reverse aftereffects of unregulated aversive stimulation>regulated aversive stimulation (contrast: [Stim_Av>Stim_RegAv]>[Fix_Av>Fix_RegAv]) occurred in the bilateral amygdala, right superior parietal lobule, right cuneus, right lingual gyrus, bilateral fusiform gyrus, left calcarine fissure, and right middle occipital gyrus (see Figure 1 and Table 1).

Reverse task-rest interactions of unregulated aversive stimulation>neutral stimulation (contrast: [Stim_Av>Stim_Neu]>[Fix_Av>Fix_Neu]) were found in the bilateral visual area V5/MT and in earlier visual areas. Representative time courses with detailed consideration of the curve shapes are depicted in Figure 1 and the full set of time courses is provided in the Supporting Information materials.

No further reverse task-rest interactions were observed. Posthoc analyses showed no significant differences in the observed reverse task-rest interactions between the emotion regulation strategies employed (see limitations section).

### 3.4 Manipulation check

Differential brain activations dependent on condition (one-sample t-test on the images of the differential first-level contrasts) were observed during stimulation, indicating a successful experimental manipulation. During unregulated aversive as compared to neutral stimulation (Stim_Av>Stim_Neu), we found increased activation in the bilateral amygdala, extensive occipital and temporal visual areas, anterior insula, motor areas, posterior parietal cortex, subcortical regions including the thalamus, cingulate cortex, medial frontal gyrus, and left precuneus, while the inverse contrast (Stim_Neu>Stim_Av) activated the left lingual gyrus as well as the right parahippocampal and angular gyri, precuneus, and orbitofrontal cortex. During the regulation condition compared to unregulated aversive stimulation (Stim_RegAv>Stim_Av), brain activation was increased in the bilateral dorsolateral prefrontal cortex, inferior parietal lobule, precuneus, and cingulate cortex as well as the right anterior insula (see Table S1). Activation during unregulated aversive stimulation as compared to regulated aversive stimulation (Stim_Av>Stim_RegAv) was greater in the bilateral amygdala, extensive occipital and temporal visual areas, superior parietal lobule, and hippocampus. For details regarding differential brain activation during fixation see Table S1.

## Discussion

### 4.1 General discussion

The goal of the present study was to test in which way induction and instructed regulation of negative emotion modulate brain activation during subsequent fixation periods. In healthy participants, similar task-rest interactions have been described for cognitively demanding tasks [6]–[7], while the modulatory effect of *affective* stimuli in task-rest switches has not previously been investigated on a whole-brain level. However, emotive stimuli have been shown to differentially impact subsequent resting-state acquisitions [2]–[3], suggesting emotion-specific task-rest interactions. These are of particular interest for psychiatric research as different patient populations characterized by pathological emotion processing show disorder-specific aberrations during the resting state [11]–[12]. Using data from an emotion regulation experiment, we were able to demonstrate concordant and reverse task-rest interactions following emotion induction as well as instructed emotion regulation. Pooling of the fMRI data was warranted by the behavioral results. The only significant group difference was found for the perceived usefulness of the instructed emotion regulation strategy which was, however, unrelated to compliance and regulation success. Despite being rated as less useful than the other strategies, expressive suppression was still perceived as moderately useful and can thus be assumed to be sufficient for a successful emotion regulation.

Concordant task-rest interactions (condition 1 activating a brain region more strongly than condition 2 during both stimulation and fixation) were observed in the dorsolateral prefrontal cortex, inferior parietal lobule and other brain regions implicated in emotion regulation. Interestingly, brain regions exhibiting a concordant task-rest interaction following regulated aversive stimulation show a large overlap with intersection regions of DMN and dorsal attention network (as extracted by Tomasi & Volkow [44]). A previously reported [25] reverse task-rest interaction (condition 1 activating a brain region more strongly than condition 2 during stimulation and vice versa during fixation) was replicated in the amygdala. Moreover, our findings also have implications for the use of fixation periods as a baseline and for the analysis of fixation periods as effects of interest in future studies.

### 4.2 Task-rest interactions: Concordant aftereffects

We defined a concordant aftereffect as an interaction between stimulation and fixation where condition 1 activates the same brain region during both stimulation and fixation more strongly than condition 2. The concordant aftereffects of regulated aversive (condition 1) greater than unregulated aversive (condition 2) stimulation in areas implicated in intentional emotion regulation were among the strongest and most widespread effects in the present analysis. Interestingly, the most dorsal portion of the right dorsolateral prefrontal cortex and the major parts of the bilateral inferior parietal lobule, posterior and middle cingulate cortex, and precuneus regions identified by this analysis are precisely at the intersection of the DMN and the dorsal attention network. In their large resting-state study (n = 979), Tomasi and Volkow [44] found the DMN to be functionally linked to a major cortical hub in the PCC/ventral precuneus and the dorsal attention network to a major cortical hub in the right IPL. The DAN has previously been implicated in saliency attribution, shifting of attention, and self-monitoring [45]. Anderson et al. [46] reported that the regions intersecting both the DMN and the dorsal attention network which are showing a concordant aftereffect in our study tend not to be anticorrelated, thus rendering a direct involvement in the switching between the two networks unlikely.

The dorsolateral prefrontal cortex and inferior parietal lobule regions exhibiting this concordant aftereffect are typically involved in top-down regulatory control of emotion, with the dorsolateral prefrontal cortex playing a crucial role in goal-directed control of attention to stimuli as well as in categorizing and evaluating feelings [10]. The middle/posterior cingulate cortex has also previously been shown to be involved in the processing and modulation of negative affect, for example in emotion regulation through cognitive reappraisal [47] or in the perception of regulated aversive social stimuli [48].

In sum, the regions exhibiting the concordant aftereffect are known to subserve the regulation of emotions. Persisting activation of these regions after stimulus offset could thus simply indicate ongoing regulatory preoccupation with the stimulus material. However, participants did not rate preoccupation with the preceding pictures differently for the two aversive fixation periods which makes it unlikely to be the cause of the observed concordant aftereffect.

The exemplary time courses for inferior parietal lobule, cingulate cortex, and precuneus (see Figure 1 and Methods Text S1) fit well with their implication in the default mode network, showing a task-negative pattern with less deactivation during emotion regulation. Notably, this difference is sustained for most of the fixation period. The exemplary dorsolateral prefrontal cortex time course for the regulated aversive condition (extracted from a ROI ventral to the intersection of DMN and dorsal attention network), on the other hand, not only shows a markedly higher signal intensity compared to the other experimental conditions but also a characteristic difference in curve shape. The continuous signal increase until stimulus offset (TR4+ca. 3 TRs delay = TR6/TR7) might reflect ongoing top-down control necessary for the sustained effortful regulation of emotion.

### 4.3 Reverse task-rest interactions

We defined a reverse task-rest interaction as an interaction between stimulation and fixation where condition 1 activates a brain region more strongly than condition 2 during stimulation while condition 2 activates the same brain regions more strongly than condition 1 during fixation. We replicated the amygdala rebound effect [25], as the bilateral amygdala exhibited a reverse task-rest interaction of greater activation during unregulated aversive stimulation as compared to regulated aversive stimulation, illustrated by the exemplary time course plots (Figure 1). The early signal intensity peak in the amygdala during unregulated stimulation (TR4) may reflect the amygdala's role as an alarm system which is suppressed during regulation. Up until the end of the stimulation, the shape and height of the amygdala time course curve during regulated aversive stimulation-fixation is very similar to that of neutral stimulation-fixation. Peaking at stimulus *offset*, 3 TRs later than the signal for unregulated aversive stimulation, this time course is unlikely to reflect a merely time-shifted direct response to the stimulation. However, the reverse task-rest interaction is also not simply a consequence of picture offset as it was only present following regulated aversive stimulation but not following any of the other conditions. It can be assumed that the amygdala rebound effect is also not a result of conscious rumination about previously down-regulated items (as there was no significant difference in the participants' preoccupation with the preceding pictures between the fixation periods following the two negative emotion conditions), but is likely an inherent characteristic of complex amygdalar interactions. As the amygdala is not a homogeneous structure but rather consists of three differentiable but complexly interconnected groups of nuclei (corticomedial, central, basolateral) that are involved in various distinct input-output loops [49]–[50], the observed task-rest interaction may be due to regulatory feedback processes within this structure which, however, we cannot resolve with our imaging parameters.

In addition, there were also reverse task-rest interactions in regions that were activated more strongly during unregulated aversive stimulation than during regulated aversive stimulation mainly in early visual areas (V1 and V2) as well as in a small postcentral cluster. Likewise, but larger in extent and effect strength, the contrast of unregulated aversive versus neutral stimulation evoked a reverse task-rest interaction in the bilateral visual area V5/MT. While task-rest interactions in visual areas were clearly related to the different experimental conditions, the present experimental design is not suitable for determining whether the observed activation modulation is specific to emotional stimulation or whether non-emotional visual stimuli triggering stronger activations would cause similar task-rest interactions. The task-rest interactions in these regions might thus be due to the known post-stimulus undershoot in visual cortex being roughly proportional to previous activation strength [4].

### 4.4 Manipulation check

Brain activation patterns evoked by the stimulation conditions are in accordance with previous studies [25] [28]–[29] [48] and models of voluntary emotion regulation [10].

Unregulated aversive stimulation activated amygdala, hippocampus, and somatosensory areas (among others) more than did regulated aversive and neutral stimulation which is in agreement with a recent meta-analytic review on the brain basis of emotion [26]. Post-scan ratings of valence, regulation success, and preoccupation with the preceding pictures during fixation indicated a successful experimental manipulation also on a behavioral level. Interestingly, significant differences in post-scan valence ratings were found between formerly regulated aversive stimuli and unregulated aversive stimuli although stimuli were closely matched for mean valence and arousal values based on standard rating provided with the IAPS [30]. This seemingly surprising finding fits well with results by Erk et al. [28] who reported a sustained down-regulation effect in the amygdala even after a 15 min delay. Crucially, both aversive conditions were rated as much more unpleasant than the neutral stimulation.

### 4.5 Differential brain activation during fixation

Dependent on the preceding task condition, four out of the six computed fixation contrasts yielded statistically significant effects. This necessitates the conclusion that fixation activations following different types of task periods should not simply be assumed to be equivalent without further testing.

Greater fixation activations following regulated aversive stimulation as compared to fixation following unregulated aversive stimulation resembled activation in the corresponding stimulation contrast with (among others) additional recruitment of the bilateral amygdala. This resulted in task-rest interactions in frontal and parietal cortex regions implicated in emotion regulation as well as in the amygdala.

Brain activation during fixation following neutral stimulation as compared to fixation following unregulated aversive stimulation being greater in bilateral middle temporal and occipital visual areas resulted in a reverse task-rest interaction in the visual cortex.

### 4.6 Limitations

Using strictly t-tests and FWE-correction, the present analyses are fairly conservative and thus only show well-ascertained effects at the cost of potentially ignoring weaker interactions by allowing more false negatives.

As the present experimental design did not include a regulated neutral condition, it does not allow us to rule out the possibility of similar task-rest interactions following non-emotional stimulation (i.e., regulated vs. unregulated neutral). Although the occurrence of such effects seems unlikely, future studies should explicitly test for aftereffects following regulated neutral stimulation. Moreover, future studies should examine potential strategy-specific differences in the observed task-rest interactions using well-powered subgroup comparisons. Posthoc analyses showing no such differences in the present study could be due to the small sample sizes of the three strategy-specific subgroups.

Gender differences in emotion regulation have been demonstrated for cognitive reappraisal [51] as well as habitual emotion regulation [52] and can thus be assumed to exist in other types of emotion regulation as well. As only female participants were included in the present investigation, gender differences were not studied. The previous findings, however, make gender differences an important topic for future research on task-rest interactions in emotion regulation.

Finally, future studies might extend the present approach to the investigation of task-rest interactions in the regulation of positive emotions. However, as pointed out in a recent and very comprehensive review by Ochsner et al. [10], studies on the regulation of positive emotion are vastly outnumbered by those examining negative emotion. This is probably due to the impact of negative emotion appearing to be greater on average than the impact of positive emotion (Baumeister, 2001, as cited in [10]) and psychiatric disorders being more often hallmarked by disturbed regulation of negative rather than positive emotion (American Psychiatric Association, 1995, as cited in [10]). Predictions regarding task-rest interactions in the regulation of positive emotions are thus speculative at best but the involvement of cognitive control regions seems likely.

While the IAPS stimuli used in this study were previously shown to be valid in the German population [31], they might not be when used in a different cultural environment. In the present study, only valence ratings were collected as a measure for the affective quality of the presented stimuli. Future studies should also collect ratings of arousal (see e.g. [53]) in order to get a more precise characterization of the experimental manipulation. Ideally, ratings for the presented pictures would be collected for each trial, that is, directly following stimulus presentation, which in principle would allow to correlate brain activation with behavioral measures. For the aim of the present investigation, however, this approach would be counterproductive as such ratings would change the very effect that is being investigated. Ratings between stimulation and fixation will confound the fixation activation with rating and motor activation while ratings following the fixation period are likely to trigger deliberation about the affective value of the stimulus in anticipation of the rating.

### 4.7 Conclusion

The fMRI investigation of task-rest switching in the induction and especially the regulation of negative emotion expands our knowledge of the inner workings of the brain by illuminating hitherto largely undescribed interaction effects (i.e., the modulation of resting brain activation by the preceding stimulation or experimental manipulation and associated activation) in brain regions implicated in generating emotions or in their regulatory control.

We found significant differential task-rest interactions, dependent on the nature of the task condition. The following patterns emerged: Firstly, various regions showed stronger activations during the fixation period following the emotion regulation condition than following the permit condition. These regions are all located at the intersection of two large-scale resting-state networks, the DMN and the dorsal attention network, and show a task-negative activation pattern. Notably, the dorsolateral prefrontal cortex signal steadily increases during the regulation task and peaks at the end of the stimulation, possibly reflecting the ongoing top-down control necessary for emotion regulation. Secondly, we replicated a paradoxical activation increase in the amygdala during the fixation period following the emotion regulation condition as previously reported by Walter et al. [25].

Establishing these novel effects lays the foundation for detailed future investigations of emotion-related task-rest interactions in healthy participants as well as psychiatric patients that are relevant for a more profound understanding of normal and pathological emotion processing.

In their seminal work, Fair et al. [13] concluded that resting blocks from cognitively or emotionally demanding experiments are well suited for resting-state analyses. However, our findings indicate that the activation during the fixation condition is differentially modulated by the preceding task-activation and thus more suitable for investigating task-rest interactions than pure resting-state. Our findings also imply that fixation periods may only be suitable as a baseline for task-related activations if experimental conditions are perfectly balanced (for further methodological implications see [8] [54]). These results also favor comparatively long resting periods that allow the signal to reach baseline. When shorter fixation periods are used, it should be considered whether the above-described modulations could carry over to the next task-activation, which would be difficult to account for with established models. More generally, the observed task-rest interactions, when replicated, will need to be taken into account also in a wide range of psychological experiments that do not rely on brain measures. This may pose a problem to standard analyses, making possible carry-over effects an important topic for further research.

## Supporting Information

Figure S1 **Reverse task-rest interaction time courses 1.** The figure shows the grand mean signal time courses (computed over blocks and participants) for regulated (red) and unregulated aversive (blue) and neutral (green) stimulation-fixation extracted from brain regions exhibiting a reverse task-rest interaction following unregulated aversive stimulation>regulated aversive stimulation. Stimulation onset is at TR1, stimulation offset is at TR 4. The activation in response to the stimulation should be expected to be delayed by about 3 TRs which corresponds to the typical lag of the canonical hemodynamic response.
(TIF)

Figure S2 **Concordant task-rest interaction time courses.** The figure shows the grand mean signal time courses (computed over blocks and participants) for regulated (red) and unregulated aversive (blue) and neutral (green) stimulation-fixation extracted from brain regions exhibiting a concordant task-rest interaction following regulated aversive stimulation>unregulated aversive stimulation. Stimulation onset is at TR1, stimulation offset is at TR 4. The activation in response to the stimulation should be expected to be delayed by about 3 TRs which corresponds to the typical lag of the canonical hemodynamic response.
(TIF)

Figure S3 **Reverse task-rest interaction time courses 2.** The figure shows the grand mean signal time courses (computed over blocks and participants) for regulated (red) and unregulated aversive (blue) and neutral (green) stimulation-fixation extracted from brain regions exhibiting a reverse task-rest interaction following unregulated aversive stimulation>neutral stimulation. Stimulation onset is at TR1, stimulation offset is at TR 4. The activation in response to the stimulation should be expected to be delayed by about 3 TRs which corresponds to the typical lag of the canonical hemodynamic response.
(TIF)

Table S1 **Effects and task-rest interactions of regulated aversive, unregulated aversive, and neutral stimulation.** The table shows anatomical labels, cluster sizes, t-scores, and coordinates in MNI space for brain activations in the contrasts of interest; threshold: p<.05, FWE-corrected. Stim_RegAv = regulated aversive stimulation; Stim_Av = unregulated aversive stimulation; Stim_Neu = neutral stimulation; Fix_RegAv = fixation following regulated aversive stimulation; Fix_Av = fixation following unregulated aversive stimulation; Fix_Neu = fixation following neutral stimulation.
(DOC)

Methods Text S1 **Examples for the modeling of task-rest interactions.** The text provides detailed examples for the GLM of a concordant task-rest interaction and the GLM of reverse task-rest interaction.
(DOC)
