# Supplementary figures and images for: The Impact of Stimulus Valence and Emotion Regulation on Sustained Brain Activation: Task-Rest Switching in Emotion
Source: PLoS One. 2014 Mar 28;9(3):e93098. doi: 10.1371/journal.pone.0093098 (PMC3969367; doi:10.1371/journal.pone.0093098)

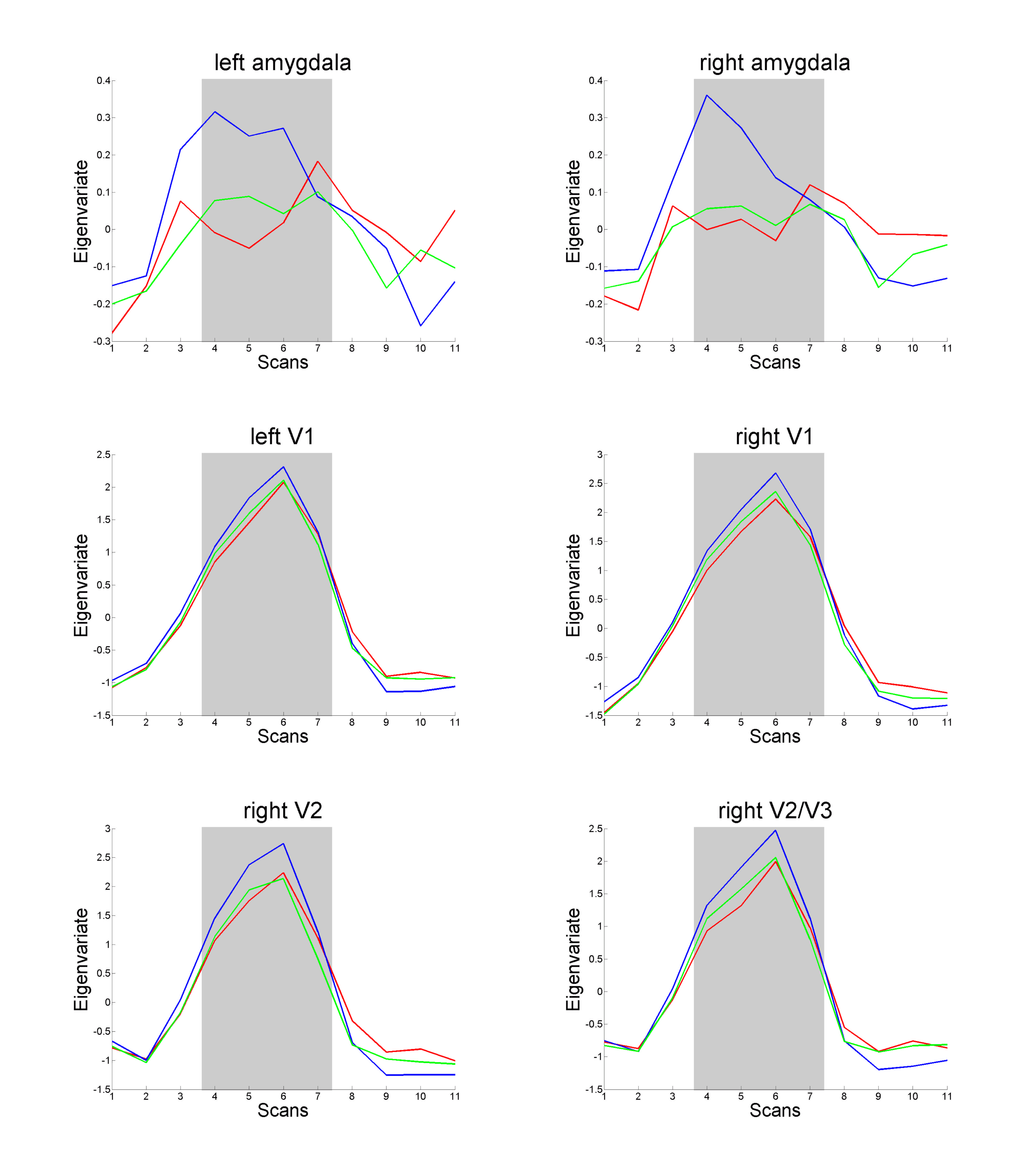

Supplement: Figure S1 — Reverse task-rest interaction time courses 1. The figure shows the grand mean signal time courses (computed over blocks and participants) for regulated (red) and unregulated aversive (blue) and neutral (green) stimulation-fixation extracted from brain regions exhibiting a reverse task-rest interaction following unregulated aversive stimulation>regulated aversive stimulation. Stimulation onset is at TR1, stimulation offset is at TR 4. The activation in response to the stimulation should be expected to be delayed by about 3 TRs which corresponds to the typical lag of the canonical hemodynamic response. (TIF) [file pone.0093098.s001.tif]

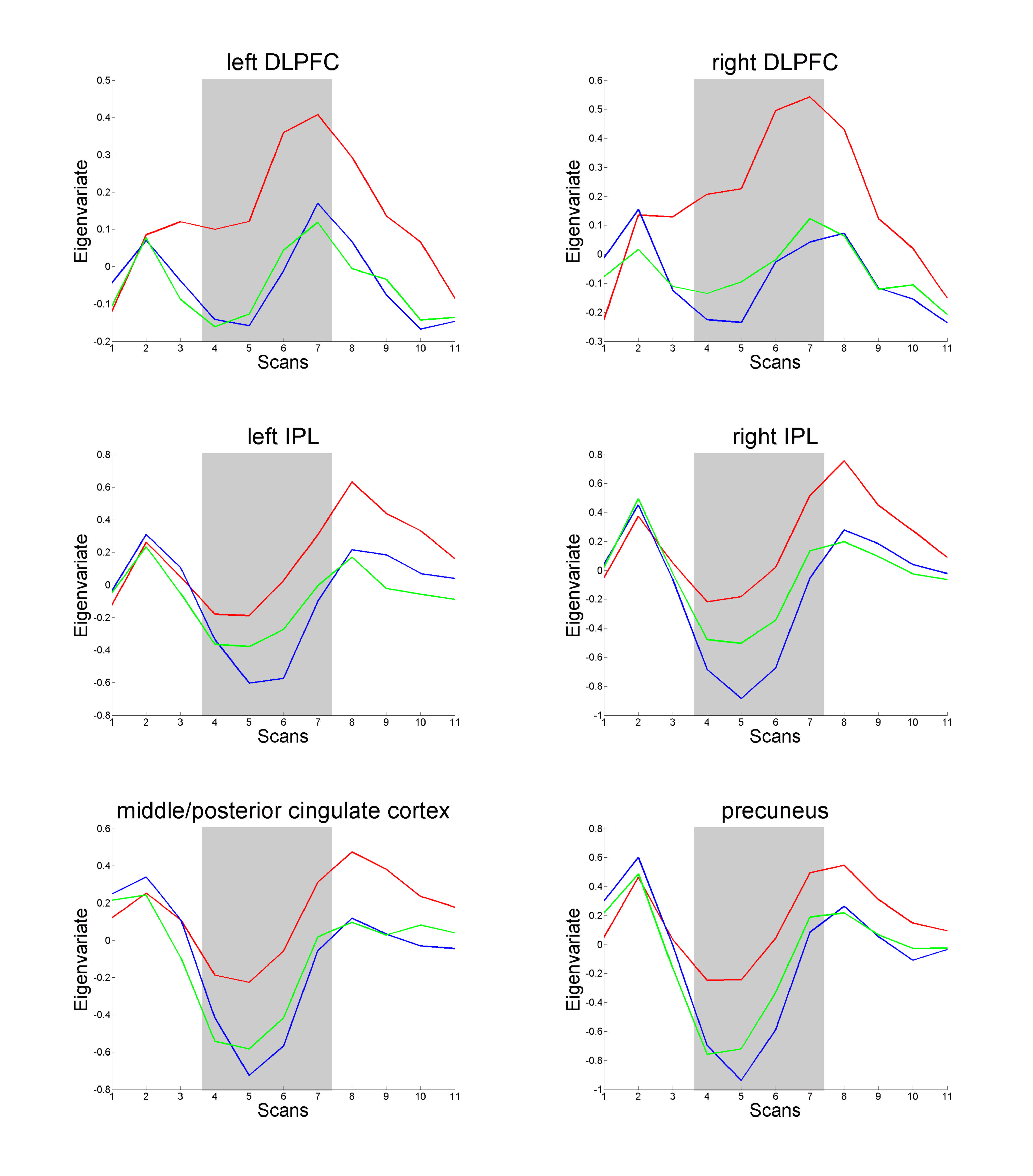

Supplement: Figure S2 — Concordant task-rest interaction time courses. The figure shows the grand mean signal time courses (computed over blocks and participants) for regulated (red) and unregulated aversive (blue) and neutral (green) stimulation-fixation extracted from brain regions exhibiting a concordant task-rest interaction following regulated aversive stimulation>unregulated aversive stimulation. Stimulation onset is at TR1, stimulation offset is at TR 4. The activation in response to the stimulation should be expected to be delayed by about 3 TRs which corresponds to the typical lag of the canonical hemodynamic response. (TIF) [file pone.0093098.s002.tif]

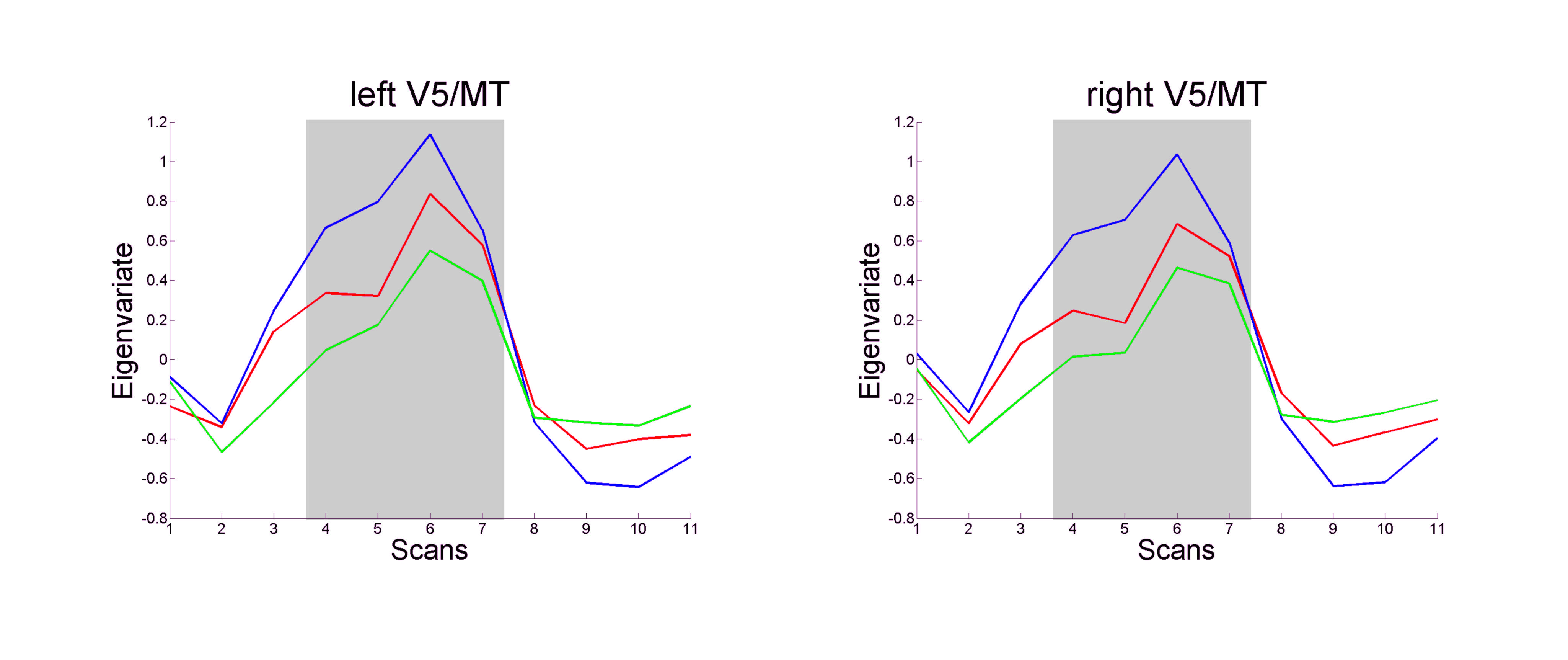

Supplement: Figure S3 — Reverse task-rest interaction time courses 2. The figure shows the grand mean signal time courses (computed over blocks and participants) for regulated (red) and unregulated aversive (blue) and neutral (green) stimulation-fixation extracted from brain regions exhibiting a reverse task-rest interaction following unregulated aversive stimulation>neutral stimulation. Stimulation onset is at TR1, stimulation offset is at TR 4. The activation in response to the stimulation should be expected to be delayed by about 3 TRs which corresponds to the typical lag of the canonical hemodynamic response. (TIF) [file pone.0093098.s003.tif]
