# Supplementary material for: The Impact of Stimulus Valence and Emotion Regulation on Sustained Brain Activation: Task-Rest Switching in Emotion
Source: PLoS One. 2014 Mar 28;9(3):e93098. doi: 10.1371/journal.pone.0093098 (PMC3969367; doi:10.1371/journal.pone.0093098)
Supplement: Table S1 — Effects and task-rest interactions of regulated aversive, unregulated aversive, and neutral stimulation. The table shows anatomical labels, cluster sizes, t-scores, and coordinates in MNI space for brain activations in the contrasts of interest; threshold: p<.05, FWE-corrected. Stim_RegAv = regulated aversive stimulation; Stim_Av = unregulated aversive stimulation; Stim_Neu = neutral stimulation; Fix_RegAv = fixation following regulated aversive stimulation; Fix_Av = fixation following unregulated aversive stimulation; Fix_Neu = fixation following neutral stimulation. (DOC) [file pone.0093098.s004.doc]

| **Contrast** | **Regions** | **Right/left** | **Cluster size**  **(voxels)** | **t-score local max.** | **MNI coordinates (x, y, z)** | | |
| --- | --- | --- | --- | --- | --- | --- | --- |
| Stim_RegAv > Stim_Av | inferior parietal lobule | R | 596 | 12.05 | 51 | -54 | 51 |
|  | middle frontal gyrus | R | 938 | 11.38 | 39 | 30 | 39 |
|  | middle frontal gyrus | R |  | 9.16 | 36 | 48 | 18 |
|  | middle frontal gyrus | R |  | 8.43 | 30 | 48 | 27 |
|  | precuneus | R | 180 | 8.56 | 12 | -63 | 36 |
|  | precuneus | L |  | 7.46 | -9 | -66 | 33 |
|  | insula | R | 71 | 7.89 | 36 | 12 | 3 |
|  | inferior parietal lobule | L | 220 | 7.86 | -45 | -60 | 51 |
|  | middle cingulate cortex | R | 152 | 7.66 | 3 | -21 | 30 |
|  | posterior cingulate cortex | R |  | 5.76 | 3 | -39 | 21 |
|  | cerebellum | L | 17 | 6.08 | -42 | -48 | -45 |
|  | supplementary motor area | L | 66 | 5.98 | -6 | 6 | 51 |
|  | supplementary motor area | R |  | 5.97 | 6 | 9 | 48 |
|  | middle frontal gyrus | L | 15 | 5.60 | -33 | 45 | 15 |
|  | middle cingulate cortex | R | 4 | 5.25 | 6 | 27 | 36 |
|  | middle orbitofrontal cortex | R | 1 | 5.22 | 33 | 51 | -9 |
| Stim_Av > Stim_RegAv | middle temporal gyrus | R | 1517 | 9.45 | 48 | -54 | -3 |
|  | inferior occipital gyrus | R |  | 7.74 | 39 | -72 | -6 |
|  | calcarine fissure | R |  | 7.47 | 24 | -84 | 3 |
|  | middle temporal gyrus | L | 1075 | 8.47 | -51 | -63 | -3 |
|  | middle occipital gyrus | L |  | 6.81 | -21 | -87 | 12 |
|  | middle occipital gyrus | L |  | 6.72 | -27 | -78 | 6 |
|  | superior parietal lobule | R | 103 | 8.38 | 27 | -48 | 57 |
|  | Amygdala | L | 129 | 8.03 | -30 | 0 | -18 |
|  | hippocampus | L |  | 7.33 | -18 | -6 | -15 |
|  | supra marginal gyrus | L | 75 | 7.05 | -63 | -24 | 39 |
|  | supra marginal gyrus | L |  | 6.85 | -57 | -27 | 33 |
|  | hippocampus | R | 71 | 6.94 | 39 | -18 | -15 |
|  | hippocampus | R |  | 6.49 | 21 | -6 | -15 |
|  | hippocampus | R | 25 | 6.91 | 27 | -27 | -3 |
|  | parahippocampal gyrus | L | 28 | 6.22 | -9 | -24 | -12 |
|  | lingual gyrus | R |  | 6.05 | 6 | -30 | -9 |
|  | postcentral gyrus | R | 10 | 5.69 | 63 | -18 | 42 |
|  | hippocampus | L | 5 | 5.61 | -24 | -24 | -9 |
|  | medial superior frontal gyrus | L | 3 | 5.48 | 0 | 66 | 21 |
|  | superior parietal lobule | L | 5 | 5.48 | -27 | -48 | 57 |
|  | lingual gyrus | L | 6 | 5.33 | -15 | -54 | -3 |
|  | superior frontal gyrus | L | 2 | 5.29 | -12 | 66 | 21 |
|  | medial superior frontal gyrus | L | 1 | 5.29 | -6 | 66 | 24 |
|  | cuneus | L | 4 | 5.26 | -12 | -87 | 30 |
|  | medial superior frontal gyrus | L | 2 | 5.19 | -6 | 63 | 30 |
|  | postcentral gyrus | L | 1 | 5.17 | -33 | -45 | 63 |
|  | calcarine fissure | R | 1 | 5.15 | 12 | -69 | 15 |
|  | calcarine fissure | L | 1 | 5.12 | -3 | -75 | 15 |
|  | amygdala (ROI) | L | 61 | 8.03 | -30 | 0 | -18 |
|  | amygdala (ROI) | L |  | 7.19 | -21 | -6 | -15 |
|  | amygdala (ROI) | R | 62 | 6.33 | 21 | -3 | -18 |
| Stim_Av > Stim_Neu | fusiform gyrus | L | 6204 | 15.49 | -39 | -72 | -15 |
|  | middle temporal gyrus | R |  | 14.06 | 51 | -66 | 0 |
|  | inferior temporal gyrus | R |  | 13.56 | 42 | -51 | -15 |
|  | supra marginal gyrus | L | 229 | 10.94 | -66 | -24 | 33 |
|  | insula | R | 2920 | 10.63 | 39 | 27 | 6 |
|  | insula | R |  | 10.41 | 39 | 27 | -3 |
|  | precentral gyrus | R |  | 10.29 | 45 | 3 | 27 |
|  | inferior frontal operculum | L | 241 | 9.12 | -42 | 3 | 24 |
|  | precentral gyrus | L |  | 6.16 | -48 | 0 | 39 |
|  | precentral gyrus | L |  | 5.59 | -45 | -3 | 51 |
|  | supplementary motor area | R | 215 | 8.37 | 9 | 6 | 60 |
|  | supplementary motor area | R |  | 7.18 | 6 | 3 | 72 |
|  | superior parietal lobule | R | 127 | 8.15 | 27 | -51 | 48 |
|  | inferior parietal lobule | L | 104 | 8.14 | -27 | -48 | 48 |
|  | anterior cinulate cortex | L | 215 | 7.30 | 0 | 18 | 24 |
|  | middle cingulate cortex | R |  | 6.29 | 6 | 15 | 39 |
|  | middle cingulate cortex | L |  | 6.10 | 0 | -3 | 36 |
|  | medial superior frontal gyrus | L | 76 | 6.57 | -6 | 66 | 24 |
|  | medial superior frontal gyrus | L |  | 6.12 | -3 | 51 | 30 |
|  | medial superior frontal gyrus | R |  | 5.65 | 6 | 69 | 12 |
|  | calcarine fissure | L | 32 | 6.41 | -15 | -72 | 9 |
|  | precuneus | L | 28 | 5.94 | -3 | -51 | 75 |
|  | calcarine fissure | R | 5 | 5.37 | 9 | -69 | 12 |
|  | amygdala (ROI) | R | 59 | 9.19 | 24 | -6 | -12 |
|  | amygdala (ROI) | L | 60 | 8.68 | -21 | -6 | -12 |
|  | amygdala (ROI) | L |  | 8.11 | -27 | 0 | -15 |
| Stim_Neu > Stim_Av | lingual gyrus | L | 18 | 6.96 | -30 | -45 | -3 |
|  | parahippocampal gyrus | R | 12 | 6.03 | 30 | -42 | -3 |
|  | precuneus | R | 5 | 5.68 | 15 | -54 | 18 |
|  | angular gyrus | R | 18 | 5.61 | 45 | -63 | 42 |
|  | middle orbitofrontal cortex | R | 9 | 5.53 | 42 | 51 | -3 |
| Fix_RegAv > Fix_Av | superior parietal lobule | R | 1048 | 8.37 | 39 | -57 | 54 |
|  | inferior parietal lobule | R |  | 7.82 | 51 | -39 | 51 |
|  | inferior parietal lobule | R |  | 7.63 | 48 | -48 | 54 |
|  | lingual gyrus | L | 924 | 8.06 | -15 | -87 | -12 |
|  | middle occipital gyrus | L |  | 7.66 | -9 | -99 | 0 |
|  | lingual gyrus | R |  | 7.52 | 9 | -90 | -6 |
|  | middle frontal gyrus | R | 2070 | 7.84 | 42 | 12 | 48 |
|  | middle frontal gyrus | R |  | 7.63 | 39 | 45 | 27 |
|  | superior frontal gyrus | L |  | 7.56 | -15 | 21 | 57 |
|  | cerebellum | L | 128 | 7.16 | -42 | -57 | -45 |
|  | cerebellum | L |  | 6.12 | -33 | -60 | -36 |
|  | cerebellum | L |  | 5.49 | -39 | -45 | -45 |
|  | middle frontal gyrus | L | 153 | 6.87 | -36 | 54 | 12 |
|  | cerebellum | R | 75 | 6.87 | 39 | -48 | -48 |
|  | cerebellum | R |  | 6.11 | 33 | -45 | -54 |
|  | middle cingulate cortex | R | 651 | 6.84 | 3 | -21 | 45 |
|  | paracentral lobule | L |  | 6.58 | 0 | -18 | 69 |
|  | middle cingulate cortex | R |  | 6.17 | 3 | -21 | 33 |
|  | inferior frontal gyrus (triangular) | L | 160 | 6.76 | -48 | 18 | 0 |
|  | superior temporal pole | L |  | 6.03 | -57 | 12 | -6 |
|  | middle temporal gyrus | R | 78 | 6.73 | 66 | -33 | -3 |
|  | cerebellum | R | 38 | 6.70 | 21 | -39 | -21 |
|  | middle temporal gyrus | L | 171 | 6.56 | -66 | -33 | -6 |
|  | middle temporal gyrus | L |  | 6.27 | -57 | -39 | 6 |
|  | middle temporal gyrus | L |  | 6.02 | -51 | -36 | -3 |
|  | inferior parietal lobule | L | 365 | 6.56 | -54 | -51 | 36 |
|  | inferior parietal lobule | L |  | 6.37 | -42 | -57 | 57 |
|  | supra marginal gyrus | L |  | 6.16 | -48 | -51 | 30 |
|  | inferior orbitofrontal cortex | L | 42 | 6.28 | -48 | 42 | -9 |
|  | inferior frontal gyrus (triangular) | L | 45 | 6.18 | -54 | 15 | 24 |
|  | cerebellum | L | 11 | 5.81 | -12 | -60 | -51 |
|  | cerebellum | L |  | 5.68 | -21 | -57 | -54 |
|  | middle temporal gyrus | R | 7 | 5.72 | 69 | -12 | -12 |
|  | insula | R | 13 | 5.68 | 39 | -3 | 9 |
|  | insula | R |  | 5.59 | 42 | -9 | 0 |
|  | cerebellum | R | 12 | 5.65 | 21 | -66 | -30 |
|  | anterior cinulate cortex | R | 15 | 5.65 | 12 | 39 | 9 |
|  | insula | R | 6 | 5.60 | 30 | 21 | -6 |
|  | cerebellum | R | 3 | 5.58 | 12 | -27 | -30 |
|  | superior occipital gyrus | L | 11 | 5.57 | -21 | -84 | 21 |
|  | hippocampus | R | 5 | 5.55 | 24 | -3 | -24 |
|  | superior occipital gyrus | R | 8 | 5.53 | 27 | -84 | 21 |
|  | cerebellum | L | 3 | 5.47 | -15 | -54 | -36 |
|  | cuneus | L | 4 | 5.43 | 0 | -93 | 30 |
|  | cerebellum | L | 4 | 5.42 | -24 | -36 | -48 |
|  | inferior temporal gyrus | R | 2 | 5.34 | 54 | -54 | -18 |
|  | superior temporal gyrus | R | 8 | 5.33 | 51 | -18 | 6 |
|  | rolandic operculum | R | 4 | 5.30 | 63 | -15 | 12 |
|  | insula | R | 3 | 5.28 | 30 | -24 | 15 |
|  | postcentral gyrus | R | 3 | 5.25 | 42 | -21 | 45 |
|  | precentral gyrus | R | 2 | 5.22 | 30 | -18 | 60 |
|  | superior temporal gyrus | R | 2 | 5.22 | 63 | -54 | 21 |
|  | supra marginal gyrus | R | 2 | 5.22 | 48 | -30 | 24 |
|  | inferior temporal gyrus | R | 1 | 5.21 | 60 | -21 | -21 |
|  | insula | R | 1 | 5.19 | 39 | 3 | -3 |
|  | lingual gyrus | R | 1 | 5.18 | 21 | -66 | -9 |
|  | postcentral gyrus gyrus | L | 2 | 5.16 | -66 | -15 | 18 |
|  | precuneus | L | 1 | 5.16 | -6 | -66 | 36 |
|  | superior temporal pole | R | 1 | 5.14 | 45 | 21 | -30 |
|  | amygdala (ROI) | R | 32 | 5.15 | 24 | -3 | -21 |
|  | amygdala (ROI) | L | 21 | 3.48 | -24 | -3 | -24 |
|  | amygdala (ROI) | L |  | 3.43 | -15 | 0 | -15 |
| Fix_Av > Fix_RegAv | precuneus | L | 1 | 5.32 | -33 | -54 | 6 |
| Fix_Av > Fix_Neu | cerebellum | L | 8 | 5.44 | -33 | -81 | -30 |
|  | inferior frontal gyrus (triangular) | R | 2 | 5.35 | 51 | 24 | 6 |
|  | cerebellum | R | 5 | 5.33 | 39 | -78 | -30 |
| Fix_Neu > Fix_Av | middle temporal gyrus | R | 67 | 6.99 | 42 | -63 | 3 |
|  | middle occipital gyrus | L | 58 | 6.77 | -45 | -72 | 6 |
|  | postcentral gyrus gyrus | L | 30 | 6.60 | -48 | -27 | 60 |
|  | inferior parietal lobule | L |  | 6.12 | -57 | -24 | 51 |
|  | superior temporal gyrus | R | 28 | 6.32 | 48 | -33 | 18 |
|  | superior occipital gyrus | R | 8 | 5.69 | 27 | -81 | 36 |
|  | middle occipital gyrus | L | 7 | 5.54 | -15 | -96 | 0 |
|  | postcentral gyrus gyrus | R | 7 | 5.53 | 51 | -24 | 60 |
|  | supplementary motor area | R | 2 | 5.43 | 9 | 0 | 45 |
|  | calcarine fissure | R | 2 | 5.22 | 15 | -90 | 6 |
|  | amygdala (ROI) | R | 4 | 3.50 | 33 | 3 | -27 |
| [Stim_RegAv >Stim_Av] > [Fix_Av >Fix_RegAv] | inferior parietal lobule | R | 386 | 13.02 | 51 | -54 | 48 |
|  | middle frontal gyrus | R | 460 | 11.66 | 39 | 48 | 18 |
|  | middle frontal gyrus | R |  | 11.36 | 39 | 30 | 39 |
|  | middle frontal gyrus | R |  | 10.11 | 33 | 42 | 33 |
|  | precuneus | R | 75 | 9.89 | 9 | -66 | 39 |
|  | precuneus | L |  | 7.98 | -3 | -72 | 42 |
|  | middle cingulate cortex | R | 82 | 9.69 | 3 | -21 | 30 |
|  | inferior parietal lobule | L | 159 | 9.45 | -42 | -60 | 51 |
|  | inferior parietal lobule | L |  | 8.25 | -54 | -54 | 42 |
|  | middle frontal gyrus | L | 9 | 7.84 | -36 | 48 | 15 |
|  | precuneus | L | 1 | 7.72 | -6 | -66 | 36 |
|  | cerebellum | L | 15 | 7.25 | -39 | -48 | -42 |
|  | middle frontal gyrus | R | 1 | 7.03 | 24 | 57 | 21 |
|  | middle cingulate cortex | R | 3 | 6.93 | 6 | 30 | 36 |
|  | inferior frontal operculum | R | 3 | 6.85 | 51 | 15 | 3 |
| [Stim_Av >Stim_RegAv] > [Fix_Av >Fix_RegAv] | superior parietal lobule | R | 11 | 9.07 | 30 | -51 | 63 |
|  | cuneus | R | 26 | 8.51 | 9 | -99 | 15 |
|  | lingual gyrus | R | 29 | 8.37 | 18 | -87 | -9 |
|  | fusiform gyrus | R |  | 7.70 | 27 | -81 | -9 |
|  | fusiform gyrus | L | 7 | 7.29 | -27 | -75 | -18 |
|  | inferior temporal gyrus | R | 2 | 7.26 | 54 | -54 | -18 |
|  | calcarine fissure | L | 16 | 7.22 | -6 | -96 | 9 |
|  | middle occipital gyrus | R | 8 | 7.07 | 27 | -84 | 18 |
|  | middle occipital gyrus | L | 2 | 6.52 | -24 | -87 | 18 |
|  | amygdala | R | 20 | 8.37 | 24 | -3 | -18 |
|  | amygdala | L | 20 | 8.16 | -30 | -3 | -21 |
|  | amygdala | L |  | 7.66 | -21 | -6 | -18 |
|  | amygdala | L |  | 5.70 | -15 | 0 | -15 |
| (Stim_Av > Stim_Neu) > (Fix_Av > Fix_Neu) | middle temporal gyrus | R | 67 | 14.86 | 51 | -66 | 0 |
|  | middle temporal gyrus | R |  | 13.41 | 45 | -60 | 9 |
|  | middle temporal gyrus | L | 58 | 13.53 | -51 | -72 | 6 |
|  | middle occipital gyrus | L |  | 12.96 | -42 | -75 | 0 |
|  | middle temporal gyrus | L |  | 12.51 | -45 | -66 | 0 |
|  | middle occipital gyrus | L | 7 | 10.20 | -15 | -96 | 3 |
|  | calcarine fissure | R | 2 | 10.13 | 15 | -90 | 3 |
|  | superior occipital gyrus | R | 8 | 9.24 | 27 | -81 | 36 |
