# Supplementary material for: The Impact of Stimulus Valence and Emotion Regulation on Sustained Brain Activation: Task-Rest Switching in Emotion
Source: PLoS One. 2014 Mar 28;9(3):e93098. doi: 10.1371/journal.pone.0093098 (PMC3969367; doi:10.1371/journal.pone.0093098)
Supplement: Methods Text S1 — Examples for the modeling of task-rest interactions. The text provides detailed examples for the GLM of a concordant task-rest interaction and the GLM of reverse task-rest interaction. (DOC) [file pone.0093098.s005.doc]

*Example for the GLM of a concordant task-rest interaction*

In order to test for the concordant task-rest interaction of [regulated aversive stimulation greater than unregulated aversive stimulation] as described above, the contrasts of [regulated aversive stimulation greater than unregulated aversive stimulation] and [fixation following regulated aversive stimulation greater than fixation following unregulated aversive stimulation] would need to be tested for interaction.

Both contrasts are modeled on the first level for each participant. On the second level, they are entered into a paired t-test (as they are correlated series of measurements). The contrasts for the paired t-test, [1 -1] or [-1 1], invert the first-level contrast that is set to -1. Thus, instead of entering the contrasts of [regulated aversive stimulation greater than unregulated aversive stimulation] and [fixation following regulated aversive stimulation greater than fixation following unregulated aversive stimulation] into the paired t-test, we inverted the fixation contrast. Accordingly, the paired t-test for the concordant task-rest interaction of [regulated aversive stimulation greater than unregulated aversive stimulation] is: [regulated aversive stimulation greater than unregulated aversive stimulation] greater than [fixation following unregulated aversive stimulation greater than fixation following regulated aversive stimulation].

Note that the mask for this contrast is the intersection map of the one-sample t-tests of [regulated aversive stimulation greater than unregulated aversive stimulation] and [fixation following regulated aversive stimulation greater than fixation following unregulated], that is, of the two contrasts which are being tested for interaction.

On the whole-brain level, masks were created from the activation in a fixation contrast masked with the activation in the corresponding task contrast. Within the amygdala, masks were created from separate left and right amygdala ROI analyses in a fixation contrast masked with the activation in the corresponding ROI analyses in the task contrast.

*Example for the GLM of a reverse task-rest interaction*

In order to test for the reverse task-rest interaction of [regulated aversive stimulation greater than unregulated aversive stimulation] as described above, the contrasts of [regulated aversive stimulation greater than unregulated aversive stimulation] and [fixation following unregulated aversive stimulation greater than fixation following regulated aversive stimulation] would need to be tested for interaction.

Both contrasts are modeled on the first level for each participant. On the second level, they are entered into a paired t-test (as they are correlated series of measurements). The contrasts for the paired t-test, [1 -1] or [-1 1], invert the first-level contrast that is set to -1. Thus, instead of entering the contrasts of [regulated aversive stimulation greater than unregulated aversive stimulation] and [fixation following unregulated aversive stimulation greater than fixation following regulated aversive stimulation] into the paired t-test, we inverted the fixation contrast. Accordingly, the paired t-test for the reverse task-rest interaction of [regulated aversive stimulation greater than unregulated aversive stimulation] is: [regulated aversive stimulation greater than unregulated aversive stimulation] greater than [fixation following regulated aversive stimulation greater than fixation following unregulated aversive stimulation].

The mask for this contrast is the intersection map of the one-sample t-tests of [regulated aversive stimulation greater than unregulated aversive stimulation] and [fixation following unregulated aversive stimulation greater than fixation following regulated], that is, of the two contrasts which are being tested for interaction. (Remark: In the present study, this contrast did not yield any significant results.)

On the whole-brain level, masks were created from the activation in a fixation contrast masked with the activation in the corresponding task contrast. Within the amygdala, masks were created from separate left and right amygdala ROI analyses in a fixation contrast masked with the activation in the corresponding ROI analyses in the task contrast.
